# Ecological differentiation and habitat unsuitability maintaining a ground beetle hybrid zone

**DOI:** 10.1002/ece3.1814

**Published:** 2015-12-15

**Authors:** Yasuoki Takami, Takeshi Osawa

**Affiliations:** ^1^ Graduate School of Human Development and EnvironmentKobe UniversityTsurukabuto 3‐11, NadaKobe657‐8501Japan; ^2^ Nanional Institute for Agro‐Environmental SciencesTsukubaIbarakiJapan; ^3^ Japan Node of Global Biodiversity Information FacilityTsukubaIbarakiJapan

**Keywords:** Ecological differentiation, geographic information system, *Ohomopterus*, reproductive isolation, species distribution model

## Abstract

Exogenous selection via interactions between organisms and environments may influence the dynamics of hybrid zones between species in multiple ways. Two major models of a hybrid zone allowed us to hypothesize that environmental conditions influence hybrid zone dynamics in two ways. In the first model, an environmental gradient determines the mosaic distribution at the boundary between ecologically differentiated species (mosaic hybrid zone model). In the second model, a patch of unsuitable habitat traps a hybrid zone between species whose hybrids are unfit (tension zone model). To test these, we examined the environmental factors influencing the spatial structure of a hybrid zone between the ground beetles *Carabus maiyasanus* and *C. iwawakianus* using GIS‐based quantification of environmental factors and a statistical comparison of species distribution models (SDMs). We determined that both of the hypothetical processes can be important in the hybrid zone. We detected interspecific differences in the environmental factors in presence localities and their relative contribution in SDMs. SDMs were not identical between species even within contact areas, but tended to be similar within the range of each species. These results suggest an association between environments and species, and provide evidence that ecological differentiation between species plays a role in the maintenance of the hybrid zone. Contact areas were characterized by a relatively high temperature, low precipitation, and high topological wetness. Thus, the contact areas were regarded as being located in an unsuitable habitat with a drier climate, where those populations are likely to occur in patches with limited precipitation concentrated. A comparison of spatial scales suggests that exogenous selection via environmental factors may be weaker than endogenous selection via genitalic incompatibility.

## Introduction

Geographic variation in environmental conditions can influence the fitness of organisms inhabiting those regions, and adaptation to a specific environmental condition can determine the distributional range of a species as well as the hybrid zone between species. Hybrid zones occur when genetically distinct groups of individuals meet and mate, resulting in at least some offspring of mixed ancestry (Barton and Hewitt 1985). Analyses of hybrid zones have shed light on ecological, behavioral, and genetic barriers for gene exchange, elucidating the mechanisms of reproductive isolation between related species and fostering our understanding of the origin of species (Harrison 1993; Arnold 1997). The spatial and temporal dynamics of hybrid zones are influenced by endogenous and exogenous selection. Endogenous selection occurs as a result of morphological, behavioral, and/or genetic incompatibilities between species (Sota and Kubota 1998; Bailey et al. 2007; Macholán et al. 2007), while exogenous selection operates via organismal adaptation to external environments (Vines et al. 2003; Dodd and Afzal‐Rafii 2004; Nosil et al. 2005). The relative importance of these two types of selection, as well as their interplay, is central to understanding the dynamics of hybrid zones (Bert and Arnold 1995; Bridle et al. 2001; Ross and Harrison 2002; Vines et al. 2003; Ruegg 2008). Exogenous selection via environmental factors is often difficult to study in species that have a wide geographic range and/or those that demonstrate cryptic ecological adaptation (Kozak et al. 2008). Thus, elucidating the nature of exogenous selection is a crucial step in hybrid zone studies.

**Figure 1:**
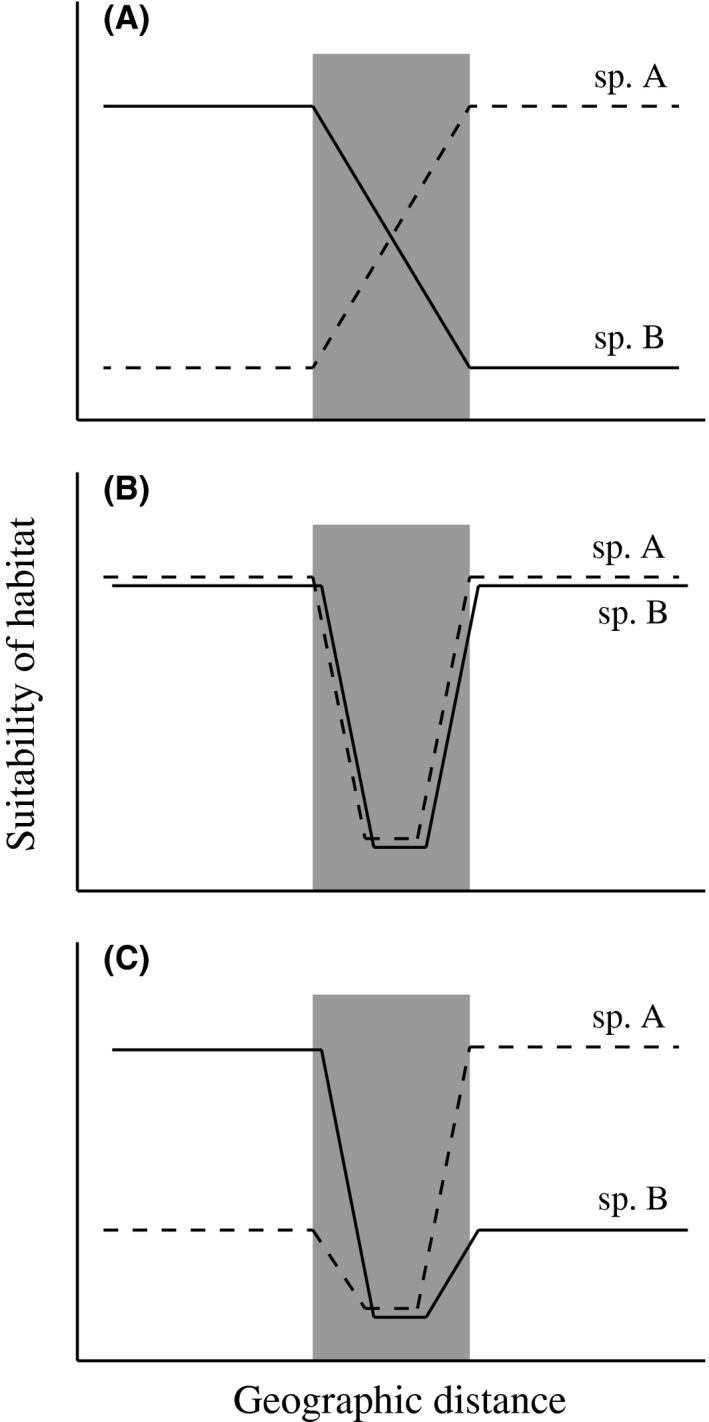
Schematic representations of exogenous selection via (A) an environmental gradient, (B) an unsuitable habitat, and (C) combination of the two in a hybrid zone (shaded area). Exogenous selection via an environmental gradient is based on ecological differentiation between species, resulting in reciprocal habitat suitability across a hybrid zone. Exogenous selection via an unsuitable habitat is based on the presence of an environment that is suboptimal for both species. Those two processes are not mutually exclusive and may mediate hybrid zones simultaneously.

The form of exogenous selection operating in hybrid zones can vary among taxa, spatial scales, and theoretical models (Ross and Harrison 2002). Two types of exogenous selection, related to two models of hybrid zones, can be distinguished. The first is selection via an environmental gradient. When two species are ecologically differentiated and adapted to different environments, and when their hybrids are unfit in the parental environment, the position of a hybrid zone is expected to coincide with an environmental gradient or boundary (i.e., ecotone) (Fig. 1A; Vines et al. 2003; Grahame et al. 2006; Ruegg 2008). This type of exogenous selection is formulated in the mosaic hybrid zone model (Harrison 1990). A mosaic hybrid zone occurs on a mosaic of environmental patches, resulting in an environment–genotype (or environment–species) association. The second type of exogenous selection occurs via an unsuitable habitat. Environments that are unsuitable for habitation by both parental species can influence the position of hybrid zones (Fig. 1B; Barton and Hewitt 1985; Tarroso et al. 2014). When hybrids are endogenously unfit, selection decreases hybrid populations, but dispersal from parental populations offsets this effect. Such source‐sink dynamics are formulated in the tension zone model (Barton and Hewitt 1985). A tension zone is a narrow cline, the width and location of which are determined by a balance between selection against hybrids and dispersal from parental species. When a patch of unsuitable habitat hinders the dispersal of one species toward the tension zone, dispersal of the other species increases, and the tension zone is pushed toward the range of the former species. This process results in the movement of the tension zone, which is finally trapped by the patch of unsuitable habitat (Fig. 1B; Barton and Hewitt 1985). These two types of exogenous selection are not mutually exclusive and are expected to cooperate in the wild (Fig. 1C), but only a few studies have detected both types in a single hybrid zone system (Tarroso et al. 2014).

Advances in geographic information systems (GIS) and techniques for species distribution modeling enable the specification of climatic and topographic factors influencing the distribution of organisms more effectively and on broader geographic scales than was previously possible (Kozak et al. 2008; Elith and Leathwick 2009). In hybrid zone studies, species distribution modeling was adopted relatively early by Kohlmann et al. (1988), but a limited number of studies have been carried out thereafter (reviewed in Swenson 2008). Most of these studies rely on graphic comparisons of model predictions to investigate niche differentiation (e.g., Chatfield et al. 2010; Engler et al. 2013; Otego et al. 2014). On the other hand, Warren et al. (2008) developed a framework for statistical comparisons of species distribution models (SDMs), enabling explicit tests of ecological or evolutionary hypotheses about the distribution of organisms. However, only a few hybrid zone studies benefited from this framework (e.g., Culumber et al. 2014).

Flightless ground beetles belonging to the subgenus *Ohomopterus* (genus *Carabus*) are a model hybrid zone system (Kubota 1988; Ishikawa 1991; Kubota and Sota 1998; Sota et al. 2000, 2001; Takami and Suzuki 2005). In a hybrid zone between *Carabus maiyasanus* and *C. iwawakianus*, a physical incompatibility between the male and female genitalia is an agent of endogenous selection (Kubota 1988; Kubota and Sota 1998; Sota and Kubota 1998). These two species are closely related (Sota and Nagata 2008; Takahashi et al. 2014) and exhibit little mate discrimination between conspecifics and heterospecifics; accordingly, interspecific mating occurs frequently (Sota and Kubota 1998). The morphologies of the male and female genitalia are highly differentiated between the species (Sasabe et al. 2010); thereby, the physical incompatibility between the heterospecific genitalia frequently injures females (Sota and Kubota 1998). This process decreases female longevity, fecundity, and fertilization success, inflicting large fitness costs to females. Additionally, the male genitalia are sometimes broken during heterospecific mating, and such males no longer mate properly (Takami 2003). Postzygotic isolation is weak between the species (Sota and Kubota 1998; Kubota et al. 2013). Accordingly, these prezygotic isolating barriers contribute to endogenous selection against hybridization, mediating narrow clines of external morphologies (i.e., tension zones) at a local scale (1–2 kilometers wide; Kubota 1988; Kubota and Sota 1998).

**Figure 2:**
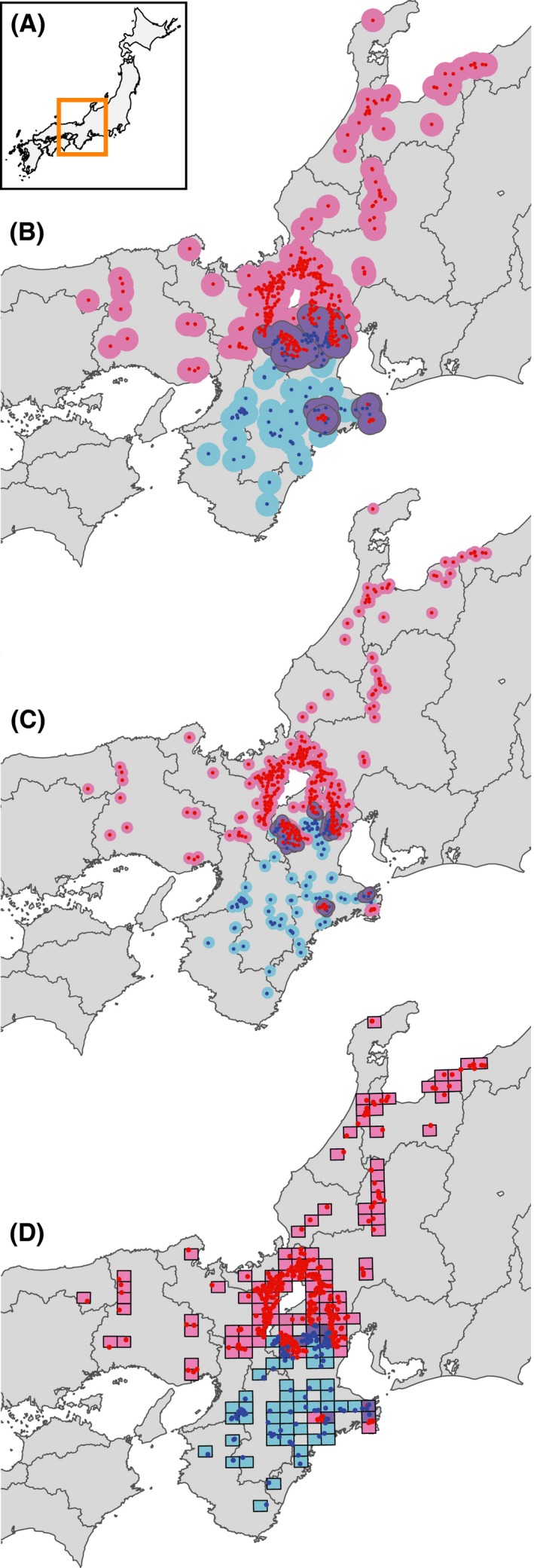
Map of presence localities of *Carabus maiyasanus* (red dots) and *C. iwawakianus* (blue dots). (A) Study area in the Japanese archipelago. Definitions of contact areas (purple) based on a (B) 10‐km buffer, (C) 5‐km buffer, and (D) 10‐km grid. Solo distributional area (pink and light blue for *C. maiyasanus* and *C. iwawakianus*, respectively) and contact area between species (light purple) are also shown.

In contrast to endogenous selection, little is known about how exogenous selection mediates the hybrid zone between *C. maiyasanus* and *C. iwawakianus* owing to their cryptic ecological differentiation. These species commonly inhabit forest floors and margins, and share prey (terrestrial invertebrates, mostly earthworms). The two species show no apparent differentiation in other life history traits (breeding season, voltinism, etc.). However, at a wide geographic scale, the contact area between the two species shows a mosaic distribution with intricate boundaries, and some populations of *C. maiyasanus* are isolated within the range of *C. iwawakianus* (Fig. 2; Ishikawa and Kubota 1994, 1995). This geographic pattern of distributional boundaries is assumed to be a footprint of past hybrid zone movement (Buggs 2007); the wide southern range of *C. maiyasanus* in the past was invaded by *C. iwawakianus* populations that moved northward. During the range expansion, the two species presumably interacted with each other and with external environments. During this process, species may colonize regions with suitable habitats and competitively exclude each other. An endogenous selection is insufficient to explain the mosaic distribution, because the dynamics of a tension zone involving a balance between endogenous selection and dispersal should minimize the length (i.e., the intricacy) of the zone (Barton and Hewitt 1985). Thus, we can reasonably hypothesize that exogenous selection via environmental factors influenced the geographic structure of the hybrid zone between *C. maiyasanus* and *C. iwawakianus*. If ecological differentiation between species plays a role in the hybrid zone, it is predicted that environmental conditions will differ between the distributional ranges of the two species, and even between two groups of patches occupied by the respective species within the contact areas. If exogenous selection via an unsuitable habitat plays a role, it is predicted that the environmental conditions in the contact areas will be relatively more severe than those in the habitats of the parental species. We characterized the environmental factors in the hybrid zone between the two ground beetle species using GIS‐based environmental quantification and species distribution modeling to test the above hypotheses of the effects of exogenous selection on hybrid zone dynamics.

## Materials and Methods

### Data acquisition

The longitude and latitude of the present species localities were compiled from multiple sources, and a total of 520 and 165 localities were identified for *C. maiyasanus* and *C. iwawakianus*, respectively. To obtain distributional data from the whole species ranges, taxonomic revisions that addressed geographic variation across the species ranges were consulted (*C. maiyasanus*:* n *=* *167, Ishikawa and Kubota 1994; *C. iwawakianus*:* n *=* *78, Ishikawa and Kubota 1995). An additional of 295 and 35 localities for *C. maiyasanus* and *C. iwawakianus* were added based on a report by the Carabidological Society of Shiga, which recorded fine‐scale distributional data within Shiga Prefecture (Carabidological Society of Shiga 2003). Shiga Prefecture is located on the boundary of two species and thus is a contact area. Additionally, several localities were identified from other studies (*C. maiyasanus*:* n *=* *24; *C. iwawakianus*:* n *=* *15; Hiura 1965; Hiura and Katsura 1971), and data were included from personal collections (*C. maiyasanus*:* n *=* *15; *C. iwawakianus*:* n *=* *18; Y. Takami, unpublished data). Hybrid populations occurring only at the boundary between the patches of the two species were easily distinguishable from parental populations by genital morphologies and were not included in the analysis. However, introgression of mitochondrial genes is extensive in this study area (Nagata et al. 2007a); thus, advanced generation hybrids with parental morphology might not have been excluded.

To avoid biases in the spatial distribution of the data, one locality was chosen randomly from a 500‐m grid when multiple species localities occurred therein. This scale was determined based on the accuracy of the environmental data set (see below). This treatment resulted in 493 and 138 localities for *C. maiyasanus* and *C. iwawakianus*, respectively.

Three environmental factors that characterize the habitats of ground beetles were examined (Antvogel and Bonn 2001): annual mean temperature (AMT), annual precipitation (AP), and topological wetness index (TWI). TWI indicates the degree of moistness at a site based on topography (a high TWI indicates that a site is moist). AMT was included in the analysis because (1) ground beetles belonging to the genus *Carabus* (s. lat.) are distributed in temperate to semi‐arctic regions in the northern hemisphere, and are thus thought to be sensitive to temperature, and (2) the two species occupy the northern and southern parts of the study area. The indices of wetness, AP, and TWI were included because the abundance of prey (earthworms) may depend on soil humidity (Richardson et al. 2009; Snyder et al. 2011). AMT and AP were obtained from 1‐km mesh meteorological values established by the Japan Meteorological Agency (2000), which is a standard meteorological data sets in Japan. The TWI was defined as ln(*a*/tan *β*), where *a* is the local upslope area that drains through a certain point per unit contour and tan *β* is the local slope value (Wilson and Gallant 2000). TWI was computed from a 50‐m digital elevation model based on 1/25,000 numerical information maps (Geospatial Information Authority of Japan 2015). These environmental layers were processed to a 500‐m grid using GIS software (ArcGIS 10.1, ESRI, Redlands, CA).

### Definition of contact areas

To compare environmental parameters and SDMs within and outside of contact areas, the localities of one species were divided into “contact” and “solo” groups according to its location near the hybrid zone or not, respectively. As the two species are essentially parapatric, a contact locality was defined as one that was in close proximity to the location of the other species. To evaluate the effect of this distance, two distance criteria, 5‐ and 10‐km, were used. These distances roughly corresponded to the maximal dispersal ability of adults over several generations based on estimates of adult movement in a reproductive season (11–14 m/day; Kubota 1996) and a reproductive period of several months in one generation. A buffer circle with a 5‐ or 10‐km radius from each locality was established. If the location of the other species was within the circle, it was considered a contact locality; otherwise, it was a solo locality. In addition to these buffer‐based definitions, a grid‐based definition was applied to account for possible bias with respect to the buffer‐based method. A grid of ca. 10‐km intervals was developed, corresponding to the Japanese Secondary Mesh unit defined by the Japanese government (hereafter, 10‐km grid), which corresponds to 1/25,000 maps. Note that the placement of this grid was independent of the distribution of the study species. If the localities of the two species co‐occurred within a cell (i.e., a 10‐km square), the localities were defined as contact, or otherwise they were defined as solo. The three definitions were called the 5‐km buffer, 10‐km buffer, and 10‐km grid. Additionally, the inner areas of the circles and cells were defined as contact or solo areas based on the classification of the localities, and these areas were used in the comparison of SDMs by background similarity tests (described below).

To evaluate the utility of the three classification schemes, the results of each were visually checked on the distributional map. Special attention was paid to how localities shaping the intricate boundaries in the central part of the study area, especially the isolated localities of *C. maiyasanus* within the range of *C. iwawakianus*, were classified. These localities should be classified as contact areas because we assumed that the populations in those localities experienced interspecific interactions and exogenous selection via environmental factors in the formation of the mosaic distribution.

### Data analysis

To characterize the environmental factors in contact areas, AMT, AP, and TWI were compared between four groups of localities (contact and solo localities of the two species). Generalized linear models (GLMs) were constructed with one of the three environmental factors as a dependent variable, and with species (*C. maiyasanus* or *C. iwawakianus*), distribution (contact or solo), and their interaction as independent variables.

SDMs were constructed for all (i.e., solo + contact) solo and contact localities of each species using Maxent ver. 3.3k (Phillips et al. 2006) with the default settings. The range of background environmental data was limited to a subset of the Japanese Primary Mesh defined by the Japanese government, which includes all of the presence localities (the area depicted in Fig. 4). Maxent predicts the occurrence of a target species based on distributional data and background environmental variables, and evaluates the probability of occurrence (i.e., environmental suitability) for each grid cell as a function of the environmental variables (Phillips et al. 2006). A logistic output of Maxent was used, with suitability values ranging from 0 (unsuitable habitat) to 1 (optimal habitat) (Phillips and Dudik 2008).

The difference between SDMs was tested based on the framework of Warren et al. (2008) using ENMTools ver. 1.3 (Warren et al. 2011). Warren et al. (2008) compared niches based on two concepts: niche equivalency, which asks whether the SDMs of related species are effectively indistinguishable (Graham et al. 2004), and niche similarity, which asks whether SDMs of related species predict one another's known occurrences better than expected under the null hypothesis that they provide absolutely no information about one another's ranges (Peterson et al. 1999). To test these, the niche identity test and background similarity test were implemented in ENMTools. Two measures of similarity between SDMs, Schoener's *D* (Schoener 1968) and Warren's *I* (Warren et al. 2008), both of which ranged from 0 (SDMs do not overlap) to 1 (SDMs are identical), were used. These similarity statistics were calculated for comparisons between (1) all localities of *C. maiyasanus* and all localities of *C. iwawakianus*, (2) contact localities of *C. maiyasanus* and contact localities of *C. iwawakianus*, (3) contact and solo localities of *C. maiyasanus*, and (4) contact and solo localities of *C. iwawakianus*. A niche identity test was performed with 500 randomizations of the identity of localities and was used to establish the null distributions of similarity statistics. The lower 5% of the null distribution was regarded as the threshold to reject the null hypothesis (i.e., one‐tailed test). The background similarity test was used to construct the null distribution of similarity statistics between the SDMs based on the localities of one species and those based on 500 replications of random points drawn from the geographic range of the other species (Peterson et al. 1999; Warren et al. 2008). The geographic range from which random points were generated was restricted to areas inside the buffer circles or cells involving presence localities because the inclusion of habitats unsuitable for both species can bias the results (Warren et al. 2008). Bidirectional tests were conducted, comparing localities of one species with random localities in the range of the other, and *vice versa*. To determine whether the distribution of one species positively or negatively predicted that of the other, the lower and upper 2.5% of the null distribution were regarded to indicate that the SDMs of the two species were significantly dissimilar and similar, respectively (i.e., two‐tailed test).

## Results

### Classification of localities

We determined that defining a contact area as a 10‐km buffer was suitable for the present data (Fig. 2), and used it in the following analyses. We observed similar results using relatively narrower scales (5‐km buffer and 10‐km grid, Fig. 2C and D), but the 10‐km grid identified a slightly smaller contact area. Both the 5‐km buffer and 10‐km grid failed to classify several *C. maiyasanus* localities isolated within the *C. iwawakianus* range as contact groups. By contrast, the 10‐km buffer captured localities around the intricate northern boundaries as contact areas, and classified the isolated populations of *C. maiyasanus* as contact localities (Fig. 2B). Using this classification, we identified 147 contact and 346 solo localities for *C. maiyasanus*, and 89 contact and 49 solo localities for *C. iwawakianus*.

### Environmental characteristics of contact areas

**Figure 3:**
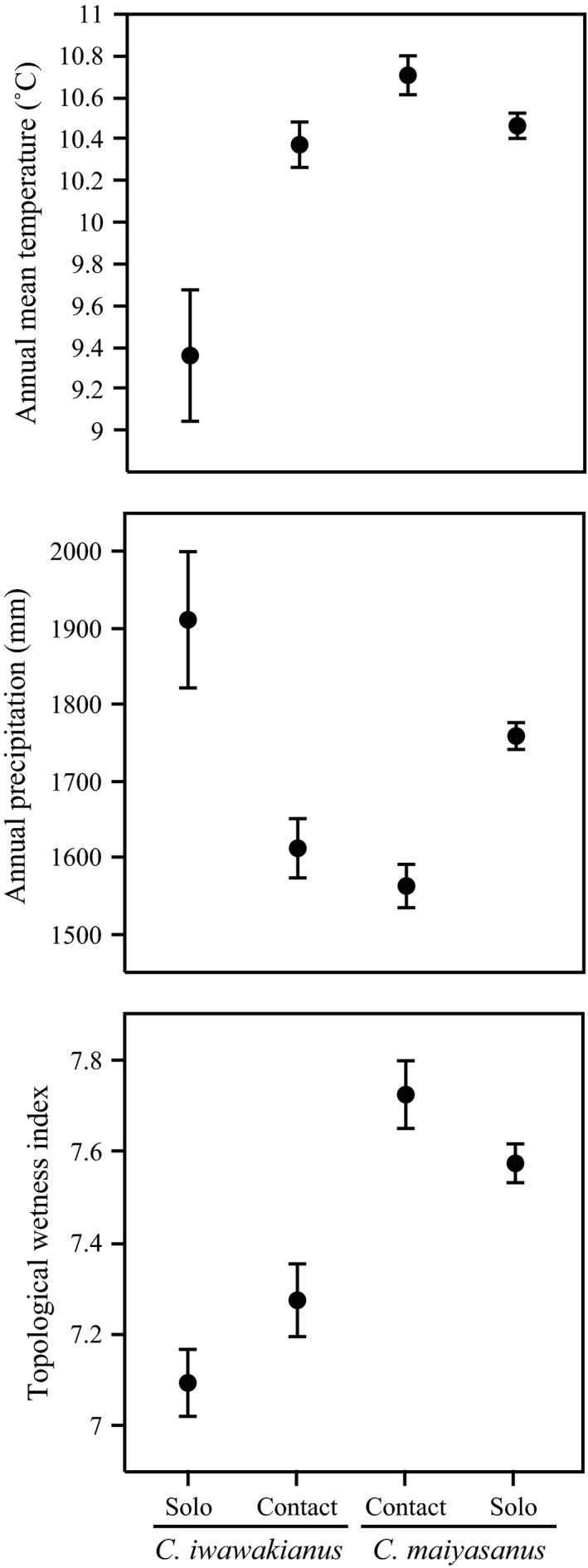
Comparisons of environmental factors between contact and solo areas in *Carabus maiyasanus* and *C. iwawakianus*.

Based on GLM analyses of environmental parameters in each of the localities, we found that the two species inhabited areas characterized by different environmental conditions, and that the conditions of the contact area were not intermediate between those of the two solo areas (Table 1, Fig. 3). AMT was significantly higher in the range of *C. maiyasanus* than in that of *C. iwawakianus* and was significantly higher in contact areas than in solo areas (Table 1, Fig. 3). The significant interaction effect indicated that the increase in AMT was greater in the contact localities of *C. iwawakianus* than in those of *C. maiyasanus* (Fig. 3). We detected a significant effect of distribution on AP, indicating that contact areas experienced significantly less rainfall and/or snowfall (Table 1, Fig. 3). We observed significantly higher TWI in the range of *C. maiyasanus* than in that of *C. iwawakianus*, and it increased significantly in contact areas in parallel (Table 1, Fig. 3).

**Table 1:** Analyses of environmental conditions in presence localities of *Carabus maiyasanus* and *C. iwawakianus*

	Statistic	*P*
Model for AMT	*F*_3, 627_ = 18.51, *R* ^2^ = 0.08	**<0.0001**
Species (*iwa*./*mai*.)	*t* = −6.20	**<0.0001**
Contact (yes/no)	*t* = 5.68	**<0.0001**
Species*contact	*t* = 3.93	**<0.0001**
Model for AP	*F*_3, 627_ = 18.15, *R* ^2^ = 0.08	**<0.0001**
Species (*iwa*./*mai*.)	*t* = 1.92	0.055
Contact (yes/no)	*t* = −6.52	**<0.0001**
Species*contact	*t* = −0.54	0.59
Model for TWI	*F*_3, 627_ = 11.51, *R* ^2^ = 0.05	**<0.0001**
Species (*iwa*./*mai*.)	*t* = −5.83	**<0.0001**
Contact (yes/no)	*t* = 1.96	**0.049**
Species*contact	*t* = 0.20	0.84

Significant effect (*P* < 0.05) was indicated in bold.

### Comparisons of the SDMs

**Figure 4:**
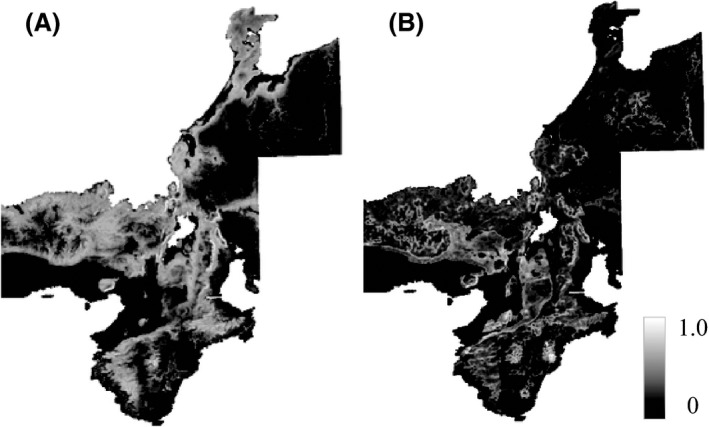
Distribution probabilities predicted by the species distribution models (SDMs) based on all localities of (A) *Carabus maiyasanus* and (B) *C. iwawakianus*. Brighter shading represents a higher probability of the distribution.

The SDMs had high performances as evidenced by the relatively high area under the curve (AUC) values (all greater than 0.78; Table 2). The contributions of environmental factors differed consistently between species; AMT and AP were relatively more important in *C. maiyasanus*, whereas AP and TWI were relatively more important in *C. iwawakianus* (Table 2). Based on the distribution probabilities predicted by the SDMs for all localities, the suitable habitat for each species was wider than the current species range, and the estimated suitable ranges largely overlapped (Fig. 4).

**Table 2:** Area under the curve (AUC) values and percent contribution of environmental variables used in MaxEnt models for occurrences of *C. maiyasanus* and *C. iwawakianus*

	*C. maiyasanus*			*C. iwawakianus*		
	All	Solo	Contact	All	Solo	Contact
AUC	0.78	0.785	0.829	0.781	0.783	0.823
Annual mean temperature (AMT)	47.5	49.2	34.4	17.8	13.2	31.4
Annual precipitation (AP)	39.8	39.1	59.2	41	52	34.8
Topological wetness index (TWI)	12.7	11.7	6.5	41.2	34.8	33.8

**Figure 5:**
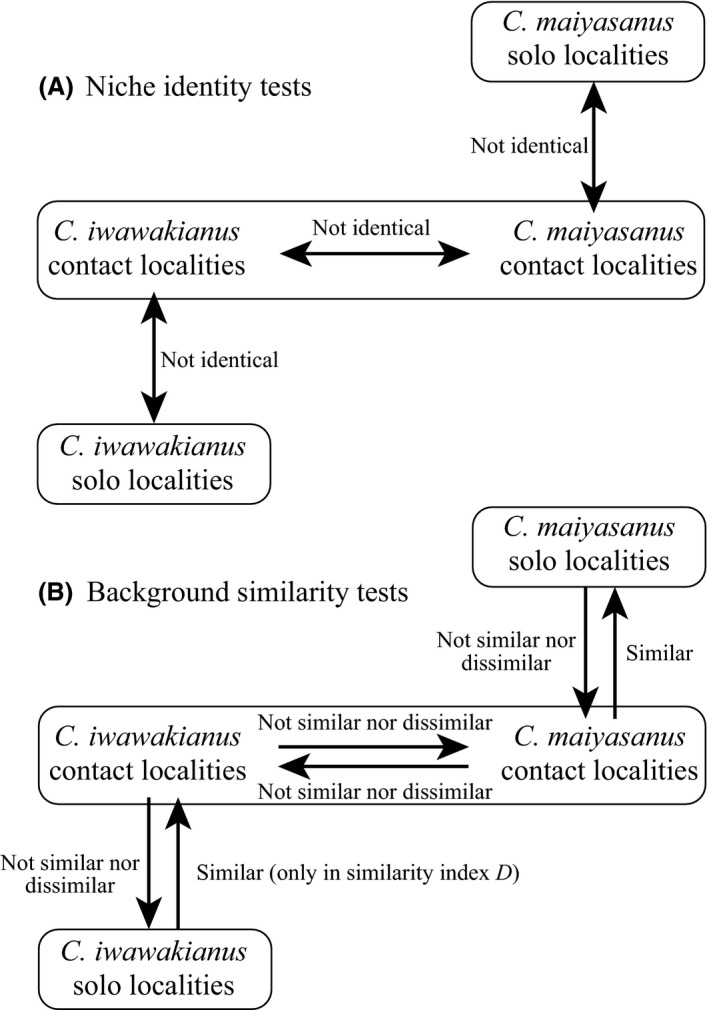
Statistical comparison of species distribution models (SDMs) by (A) niche identity tests and (B) background similarity tests.

Based on niche identity tests, we determined that the SDMs were not identical between species for all localities and for contact localities (Table 3). We detected differences in SDMs within each species, that is, between solo and contact localities (Table 3, Fig. 5A).

**Table 3:** Niche identity test results. Similarity statistics and summary of null distributions (lower 0%, 2.5%, and 5% values) are shown

Comparison	Similarity statistics	Null distribution		
		0%	2.5%	5%
*C. maiyasanus* (all) versus	*D* = 0.732**	0.777	0.820	0.823
*C. iwawakianus* (all)	*I* = 0.812**	0.838	0.867	0.869
*C. maiyasanus* (contact) versus	*D* = 0.760**	0.735	0.781	0.788
*C. iwawakianus* (contact)	*I* = 0.825**	0.815	0.841	0.848
*C. maiyasanus* (contact) versus (solo)	*D* = 0.684**	0.794	0.853	0.863
	*I* = 0.768**	0.837	0.881	0.890
*C. iwawakianus* (contact) versus (solo)	*D* = 0.723**	0.767	0.829	0.836
	*I* = 0.797**	0.842	0.874	0.883

**P < 0.002, based on 500 randomizations (one‐tailed).

Background similarity tests indicated that the SDM of *C. iwawakianus* based on all localities positively predicted that of *C. maiyasanus* based on all localities, but not in the opposite direction (Table 4). Within the contact area, the SDM of *C. iwawakianus* did not predict that of *C. maiyasanus* and *vice versa* (Table 4, Fig. 5B). We observed similar SDMs within each species. The SDM of *C. iwawakianus* based on solo localities positively predicted that based on contact localities, only in the similarity statistic *D*, but not in the opposite direction; the SDM of *C. maiyasanus* based on solo localities also positively predicted that based on contact localities, but not in the opposite direction (Table 4, Fig. 5B).

## Discussion

Most studies on exogenous selection operating in hybrid zones have focused on ecological differentiation along environmental gradients (e.g., Harrison 1990; Vines et al. 2003; Grahame et al. 2006). Relatively few studies have reported evidence of unsuitable habitats at boundaries (e.g., Tarroso et al. 2014), although they have been predicted theoretically (Barton and Hewitt 1985). Our results revealed that these two types of exogenous selection can operate together in a single hybrid zone system. Additionally, most hybrid zone studies in *Ohomopterus* ground beetles focused on mitochondrial introgression and the mechanisms of endogenous selection responsible for reproductive isolation between species (Kubota 1988; Kubota and Sota 1998; Sota and Kubota 1998; Sota et al. 2000, 2001; Takami and Suzuki 2005; Ujiie et al. 2005; Usami et al. 2005; Takami et al. 2007; Nagata et al. 2007a,b; Kubota et al. 2013), but no study has examined exogenous selection via environmental factors (cf., Tsuchiya et al. 2012). This is the first study to examine the hypothesis that environmental factors influence the dynamics of a hybrid zone of *Ohomopterus* ground beetles. As our results are solely based on a comparison of habitat environmental factors, additional examinations such as common garden experiments, transplantation, and population genetic analyses will be necessary for a more rigorous determination of the dynamics of the hybrid zone.

The observed association between environments and species suggests that ecological differentiation between species played a role in the formation of a mosaic distribution in the contact area. The environment–species association was evidenced by (1) the difference in environmental factors between species localities (Table 1, Fig. 3); (2) the consistent difference in the relative contribution of the three environmental factors in SDMs between species (Table 2); and (3) the difference between the SDMs of the two species even within contact areas, despite the shared background environment (Table 3, Fig. 5A). However, the degree of ecological differentiation between species was not large, as indicated by the ability of the SDM of *C. iwawakianus* to predict that of *C. maiyasanus*. In turn, niches tended to be conserved within species, as indicated by the similarity between the SDMs of contact and solo areas in each species (but only in one direction, Table 4, Fig. 5B). Collectively, we detected weak but constant niche differentiation between species, providing support for the hypothesis that exogenous selection via an environmental gradient influences hybrid zone dynamics. However, we also uncovered some inconsistencies; for instance, the results of the background similarity tests depended on the direction of the test (Table 4, Fig. 5B), suggesting that the results may not be useful for deducing the process in the wild. This may be due to unequal sample sizes between the two groups of localities, and/or an uneven distribution of localities and/or environmental conditions within distributional areas (Warren et al. 2011). Thus, a more balanced and intensive sampling of localities and environmental variables could help better define the role of exogenous selection determined by an environmental gradient in the hybrid zone.

**Table 4:** Background similarity test results. Similarity statistics and summary of null distributions (lower 0%, 2.5%, 97.5%, and 100% values) are shown

Comparison	Similarity statistics	Null distribution			
		0%	2.5%	97.5%	100%
*C. maiyasanus* (all)	*D* = 0.732	0.671	0.678	0.747	0.764
*‐> C. iwawakianus* (all)	*I* = 0.812	0.782	0.783	0.824	0.834
*C. iwawakianus* (all)	*D* = 0.732*	0.684	0.687	0.727	0.735
‐> *C. maiyasanus* (all)	*I* = 0.812*	0.777	0.778	0.810	0.814
*C. maiyasanus* (contact)	*D* = 0.760	0.657	0.686	0.771	0.790
*‐> C. iwawakianus* (contact)	*I* = 0.825	0.773	0.788	0.846	0.860
*C. iwawakianus* (contact)	*D* = 0.760	0.714	0.738	0.802	0.818
‐> *C. maiyasanus* (contact)	*I* = 0.825	0.793	0.810	0.852	0.863
*C. maiyasanus* (contact)	*D* = 0.684**	0.512	0.536	0.581	0.596
‐> (solo)	*I* = 0.768**	0.682	0.694	0.717	0.726
*C. maiyasanus* (solo)	*D* = 0.684	0.658	0.679	0.740	0.760
‐> (contact)	*I* = 0.768	0.751	0.767	0.807	0.817
*C. iwawakianus* (contact)	*D* = 0.723	0.594	0.626	0.730	0.758
*‐>* (solo)	*I* = 0.797	0.713	0.739	0.802	0.823
*C. iwawakianus* (solo)	*D* =* *0.723*	0.622	0.651	0.720	0.736
*‐>* (contact)	*I* = 0.797	0.743	0.759	0.800	0.806

Directions of tests are indicated by arrows (presence localities of one species ‐> geographic range of the other species in which random points were generated).

**P < 0.004 and *P < 0.05, based on 500 randomizations (two‐tailed).

Ecological differentiation between species may result from a latitudinal gradient of environmental factors and topographic characteristics in the region. Although AP did not differ between the ranges of the two species (Table 1, Fig. 3), the northern area of the *C. maiyasanus* range experiences relatively heavy snowfall in the winter, while the southern area of the *C. iwawakianus* range receives relatively heavy rainfall in the summer (Japan Meteorological Agency 2000). The relatively low AMT and TWI in the *C. iwawakianus* range suggest that this species inhabits areas of high elevation and ridge‐like regions (Figs. 3 and 4). A geographic barrier related to the median tectonic line running latitudinally at the southernmost part of the contact area may also promote genetic differentiation between species. Thus, the two species are assumed to adapt to different climates along a background environmental gradient, and this may facilitate colonization in patches suitable for their own ecological requirements within the contact area. Phylogeographic analyses using molecular markers will be necessary to examine these hypotheses of species differentiation and colonization history.

Based on a GIS‐based evaluation of environmental factors in species localities, we determined that the contact areas are situated in an unsuitable habitat. The environmental conditions in the contact areas were not intermediate between those of the solo areas of each species; instead, they comprised a distinct set of environmental conditions, including a high AMT, low AP, and high TWI (Fig. 3). With respect to topography, the localities in the contact area are relatively low and flat. From a biological point of view, the relatively high AMT and low AP observed in the contact localities indicate that the two species are in contact in drier climatic regions. The relatively high TWI observed in the contact localities may reflect a compensatory reaction of the beetle populations, that is, populations are likely to occur in places where a limited amount of precipitation is concentrated on the ground, which generates relatively wet patches within the dry climatic region. However, the degree to which dryness in the contact areas is offset by high topological wetness is unclear. Alternatively, populations in contact areas might benefit from dry conditions with high temperatures and low precipitation levels and prefer to avoid the excessive humidity associated with high TWI. It will be necessary to determine the optimal temperature and humidity conditions for beetle fitness to discriminate between these two possibilities. Generally, however, high environmental wetness, especially high soil humidity, is one of the most crucial habitat conditions for ground beetles (Antvogel and Bonn 2001) because the abundance of earthworms, the specific larval prey of the two species, is also sensitive to soil humidity (Richardson et al. 2009; Snyder et al. 2011). Thus, our results can be interpreted to indicate that the environment in contact localities is more severe with respect to wetness than the environment in solo localities. The results are consistent with the hypothesis that exogenous selection via an unsuitable habitat influences hybrid zone dynamics.

Another type of exogenous selection may influence species in hybrid zones, but it is not consistent with the present system. The bounded superiority model of hybrid zones (Moore 1977) predicts that hybrid populations are adapted to the environments of contact areas. However, this is unlikely because the species are essentially parapatric, and there is no stable hybrid population within narrow clines between the species ranges (Kubota 1988; Kubota and Sota 1998). This type of exogenous selection may occur in the hybrid zone between the congeneric species *C. arrowianus* and *C. insulicola* (Sota et al. 2000; Ujiie et al. 2005). These two species inhabit and are in contact in the river basins segmented by tributaries, establishing hybrid swarms therein. The hybrid swarms are probably maintained by limited dispersal from parental populations on the upper and lower sides of the rivers, and by endogenous and/or exogenous selection operating in the hybrid populations (Sota et al. 2000).

The geographic scale on which endogenous and exogenous selection pressures operate varies (Bridle et al. 2001; Ross and Harrison 2002; Ross et al. 2008), and this variation has implications for their relative contributions to reproductive isolation. The geographic scales of endogenous and exogenous selection are concordant in some hybrid zones (Bridle et al. 2001; Grahame et al. 2006), but discordant in others (Ross and Harrison 2002; Ross et al. 2008). A narrower scale indicates that selection operates more locally and more strongly against hybridization because stronger selection eliminates individuals with intermediate phenotypes from contact areas more intensively and allows parental individuals to meet and interact in closer proximity (Barton and Hewitt 1985). The level of environmental heterogeneity, or the steepness of an environmental gradient, may have a similar influence on the scale and strength of exogenous selection. In the hybrid zone between *C. maiyasanus* and *C. iwawakianus*, the scale of the mosaic distribution in the contact area (several tens of kilometers in width, Fig. 2) was much broader than the scale of morphological clines resulting from endogenous selection via morphological incompatibility of the genitalia (1–2 km; Kubota 1988; Kubota and Sota 1998). Weak ecological differentiation, or sparse ecological heterogeneity, may be responsible for the observed scale over which exogenous selection operates. These findings suggest that reproductive isolation via exogenous selection is not of primary importance in *C. maiyasanus* and *C. iwawakianus* speciation. Alternatively, endogenous selection via morphological incompatibilities between heterospecific genitalia may play a principal role (Sota and Kubota 1998). The role of the endogenous selection is also suggested by the distribution predicted by the SDMs of the two species (Fig. 4), which overlapped broadly despite their essentially parapatric observed distribution (Fig. 2). These results suggest that endogenous selection via reproductive interference facilitated competitive exclusion (Okuzaki et al. 2010). Quantification of reproductive isolation via ecological differentiation (e.g., Ramsey et al. 2003) and an estimation of their relative contribution to total isolation (Takami et al. 2007; Kubota et al. 2013) are warranted to improve our understanding of speciation in *Ohomopterus* ground beetles.

## Conflict of Interest

The authors declare no conflict of interest.
